# Chemical Synthesis and Characterization of Lanthanum(III) 2‑Hydroxybenzoate and Its Antibacterial Potential: *In Vitro* and *In Silico* Approaches

**DOI:** 10.1021/acsomega.6c02200

**Published:** 2026-06-08

**Authors:** Okan Aykaç, Kerim Emre Öksüz, Zeynep Sümer

**Affiliations:** † Department of Pharmaceutical Chemistry, Institute of Health Sciences, Inönü University, Malatya 44280, Türkiye; ‡ Department of Pharmaceutical Chemistry, Faculty of Pharmacy, 52954 Sivas Cumhuriyet University, Sivas 58140, Türkiye; § Department of Metallurgical and Materials Engineering, Faculty of Engineering, Sivas Cumhuriyet University, Sivas 58140, Türkiye; ∥ Department of Bioengineering, Institute of Science and Technology, Sivas Cumhuriyet University, Sivas 58140, Türkiye; ⊥ Department of Medical Microbiology, Faculty of Medicine, Sivas Cumhuriyet University, Sivas 58140, Türkiye

## Abstract

A novel lanthanum­(III) salicylate complex (La­(Sa)_3_) with potential biomedical applications was chemically synthesized via the reaction of salicylic acid (SA) with lanthanum oxide nanoparticles (La_2_O_3_NPs). This two-step synthesis approach involved the initial preparation of La_2_O_3_NPs followed by their coordination with mixed SA ligands, after which the resulting complex and its precursors were comprehensively characterized. The structure of the La­(Sa)_3_ complex was investigated using nuclear magnetic resonance (NMR) spectroscopy, Fourier transform infrared (FTIR) spectroscopy, field-emission scanning electron microscopy (FE-SEM), and transmission electron microscopy (TEM). Molecular docking studies of the newly synthesized complex were conducted to elucidate the interactions between La­(Sa)_3_ and the target microbial protein at the molecular level. Comparative evaluations of the bacteriostatic and bactericidal activities of the La­(Sa)_3_ complex, its ligand (SA), and La_2_O_3_NPs were performed against Gram-positive and Gram-negative bacteria. These studies demonstrated that the La­(Sa)_3_ complex exhibited significant antibacterial activity against the selected bacterial strains. The results further indicated that La­(Sa)_3_ showed enhanced antibacterial properties due to a synergistic effect with La_2_O_3_NPs. The morphology of the La­(Sa)_3_ complex revealed that the chemical synthesis of the La­(Sa)_3_ complex in the presence of La_2_O_3_NPs resulted in notable differences in powder and particle morphology. Spectral analyses confirmed that the synthesized La_2_O_3_NPs successfully reacted with SA to form La­(Sa)_3_ complex molecules. The data obtained from the *in silico* studies were consistent with the results of the experimentally conducted antibacterial assays. The experimental study results suggest that the La­(Sa)_3_ complex may be developed as a promising antibacterial drug candidate.

## Graphical abstract

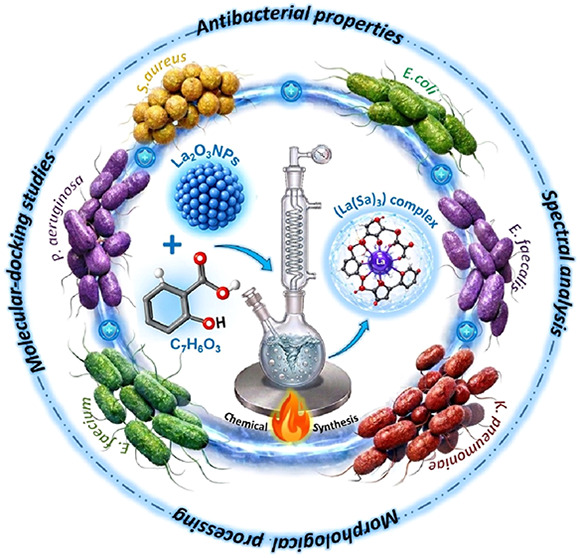

## 1 Introduction

Inorganic small molecules have long been employed in the treatment of various diseases, and numerous studies are currently underway to explore their potential for further development.^1^ Metal compounds such as Cu- and Au-based formulations have been used therapeutically since ancient times, and with the advent of modern functional medicines, platinum and its derivatives have become key agents in cancer therapy. Therefore, the therapeutic potential of inorganic compounds warrants serious consideration in developing new diagnostic and treatment strategies.^2,3^ Accordingly, research on biologically active metal-based compounds has increased significantly in recent years,^4^ although the fundamental mechanisms of action of many such compounds remain insufficiently understood.^5,6^

Infections caused by pathogenic microorganisms continue to role a substantial global health challenge despite significant advances in modern medicine. Particularly in intensive care and infectious disease settings, common bacterial species such as *S. aureus* and *E. coli* can give rise to severe and drug-resistant infections, even though they belong to different bacterial classes.^7^ The growing prevalence of antimicrobial resistance mechanisms limits the effectiveness of existing therapeutic options and underscores the urgent need for alternative strategies.^8^ Therefore, the antimicrobial potential of transition metal–containing compounds has garnered increasing interest, with various metal and metal oxide complexes emerging as promising candidates for next-generation therapeutic agents.^9–11^

Recent studies in the literature indicate that the incorporation of resveratrol into synthesized silver (Ag) nanoparticles has been investigated *in vitro*, and the results suggest that such systems may play an effective role in breast cancer therapy.^12^ *In vivo* experiments conducted with boron-doped calcium phosphate–based Ca_3_(PO_4_)_2_ graft biomaterials have demonstrated rapid and efficient healing in traumatic defects along mandibular fracture lines.^13^ Zinc oxide (ZnO) nanoparticles synthesized via the sol–gel method was incorporated into biocompatible polymers to produce hydrogels with high swelling capacity. Their physicochemical properties were further modified with amoxicillin, and their antimicrobial mechanisms against *E. coli* and *S. aureus* were characterized.^14^ Studies on Au complexes, a transition-metal–based class of compounds, have shown their potential use in the treatment of human diseases such as bacterial infections and parasitic infestations.^15^ A recent study^16^ evaluated the physicochemical and biological properties of boron-doped hazelnut-shell biochar prepared with varying concentrations of boric acid (H_3_BO_3_). The materials cytocompatibility, hemocompatibility, and antibacterial activity against *S. aureus* and *C. albicans* were subsequently assessed, yielding significant results.

Unlike transition metals such as Cu, Ag, and Au, lanthanum (La) has long been considered a largely biologically inert element.^17^ Consequently, La-containing compounds have found therapeutic applications in the treatment of certain diseases. For instance, lanthanum carbonate (La_2_(CO_3_)_3_) is a potent enteric phosphate binder employed in the management of hyperphosphatemia associated with chronic kidney disease. In the gastrointestinal tract, it ionizes to the La^3+^ form and forms insoluble lanthanum phosphate complexes with dietary phosphate, thereby inhibiting intestinal phosphate absorption. These complexes are excreted in the feces without systemic absorption, leading to reduced serum phosphate levels and contributing to clinical improvement by slowing the progression of mineral-bone disorders.^18,19^ Furthermore, La and lanthanum oxide (La_2_O_3_)-based nanoparticles are being investigated as next-generation therapeutic agents for glioblastoma (GBM). Recent studies have demonstrated that GBM cells accumulate La^3+^ ions at a higher rate than healthy astrocyte cells, a biochemical property that supports the evaluation of La as a tumor-selective agent. Additionally, La_2_O_3_ nanoparticles possess the ability to cross the blood-brain barrier (BBB), overcoming a significant obstacle in brain tumor treatment. Lanthanum’s radiosensitizing properties (Auger effect), arising from its unique electronic configuration (*f*-electropositive metallic element), combined with its capacity to synergize with chemotherapeutic agents, enhance treatment efficacy by promoting apoptosis. Therefore, La-based nanoparticles are considered promising adjuvant agents for use in combination with radiotherapy and Temozolomide (C_6_H_6_N_6_O_2_) in GBM management.^20^ Moreover, La-based nanoparticles, such as La_2_O_3_,^21^ (La­(OH)_3_), and (LaF_3_),^22^ possess distinctive properties, including high surface area, chemical stability, and the ability to generate reactive oxygen species (ROS), all of which have been reported to contribute to their antimicrobial activity.

Recent studies indicate that La compounds possess significant potential as candidates for the development of new drug delivery systems. Moreover, the latest research has demonstrated that La participates in biochemical pathways^23^ and exhibits notable biological activity.^24^ The pronounced biological activity of La compounds is attributed to the unique coordination properties of the La element.^25^ Its high coordination number contributes to the structural versatility of La-based compounds. Research on La-based complexes remains limited compared with other transition-metal systems,^26^ and their biological properties are still poorly understood, indicating the need to investigate their activity and mechanisms of action.

The biological activity of lanthanum complexes largely depends on the selection of an appropriate ligand. Compounds containing oxygen-based functional groups are still widely used in the synthesis of complexes with rare-earth elements. In this study, salicylic acid (SA, C_7_H_6_O_3_), a pharmacological agent well-known for its analgesic, antioxidant, and anti-inflammatory properties,^27^ was selected as the ligand, and a new La_2_O_3_ based complex was prepared to investigate its potential synergistic effects. In addition to experimental investigations, *in silico* approaches have become an indispensable tool in modern drug discovery and development processes, particularly for elucidating molecular-level interactions between bioactive compounds and their biological targets.^28,29^ In this paper, molecular docking simulations were performed to elucidate the interaction of the synthesized lanthanum salicylate (La­(Sa)_3_) complex with a representative microbial target protein. This *in silico* analysis was conducted to support the *in vitro* antibacterial findings by evaluating the binding behavior of the complex and examining the possible mechanistic contribution of lanthanum coordination to the enhanced biological activity observed.

## 2 Experimental Study

### 2.1 Materials and Chemicals

Lanthanum­(III) oxide powders (La_2_O_3_, ≥99.9% purity), Salicylic acid (Sa, 2-Hydroxybenzoic acid, ACS reagent grade, ≥ 99.0%), *N,N* Dimethylformamide, (DMF, HCON­(CH_3_)_2_), ACS reagent, ≥99.8%), Deuterochloroform (CDCl3, Chloroform-d, 99.8 atom % D for analyses) and Ethyl alcohol, pure ((CH_3_CH_2_OH), ≥99.5%, ACS reagent, 200 proof) were sourced from Sigma-Aldrich (St. Louis, USA). The reference microbial strains *Staphylococcus aureus* (Gram-positive, ATCC 29213), *Enterococcus faecalis*, (Gram-positive, ATCC 29212), *Enterococcus faecium* (Gram-positive, ATCC 1299), *Escherichia coli*, (Gram-negative, ATCC 25922), *Pseudomonas aeruginosa* (Gram-negative, ATCC 27853), *Klebsiella pneumonia* (Gram-negative, ATCC 700603) were purchased from the American Type Culture Collection, (Manassas, Virginia, USA). Both microorganisms were maintained as frozen stocks at – 80 °C in 20% glycerol. Muller Hinton Broth and Muller Hinton agar and all other reagents and supplies used for *in vitro* experiments were of analytical grade and procured from Merck KGaA (Darmstadt, Germany), Thermo Fisher Scientific (Massachusetts, USA), and Bayer AG (Leverkusen, Germany). Ultrapure water (dH_2_O) was prepared by using a Milli-Q50 SP reagent water system (Millipore Corporation, MA, U.S.A.) for the synthesis of all samples.

### 2.2 Chemical Synthesis and Experimental Studies

The received La_2_O_3_ powders was subjected to high-energy wet grinding in CH_3_CH_2_OH using a Pulverisette 7 Premium line planetary micromill (Fritsch GmbH, Germany, Idar-Oberstein) with a zirconium oxide (ZrO_2_) grinding bowl and 10 mm and 3 mm grinding ZrO_2_ balls at a rotation speed of 600 rpm for 1 h. The CH_3_CH_2_OH was evaporated, and the La_2_O_3_ nano particles (La_2_O_3_NPs) as a dry sample was obtained. After that the synthesis of lanthanum salicylate (La­(Sa)_3_) complex (Figure 1) was conducted using a stoichiometric ratio of three equivalents of salicylic acid (SA, C_7_H_6_O_3_) to one equivalent of La_2_O_3_NPs. The quantities of the reagents required for the reaction were calculated based on their respective molar masses. The molar mass of C_7_H_6_O_3_ is 138.12 g/mol, and the molar mass of La_2_O_3_ is 325.81 g/mol. Accordingly, 3.1 mmol of C_7_H_6_O_3_ (0.43 g) was used stoichiometrically for 1 mmol of La_2_O_3_NPs (0.33 g) in the experimental studies. The measured amount of C_7_H_6_O_3_ was refluxed in an appropriate volume of *N,N*-dimethylformamide (DMF) at 200 °C for 3 h under a reflux condenser. In a separate vessel, one equivalent of La_2_O_3_NPs was suspended in DMF using an ultrasonic homogenizer (SONICS, VCX/750, Ultrasonic Processors, Newtown, USA). Subsequently, the C_7_H_6_O_3_ solution was added dropwise to the prepared La_2_O_3_NPs suspension.^11,30^ The resulting solid product was washed sequentially with DMF and dH_2_O to remove impurities and then dried in a vacuum oven (9 × 10^–1^ bar pressure) at 50 °C for 72 h. The dried La­(Sa)_3_ complex were subsequently stored in a desiccator for use in characterization and biological activity studies.

**Figure 1:**
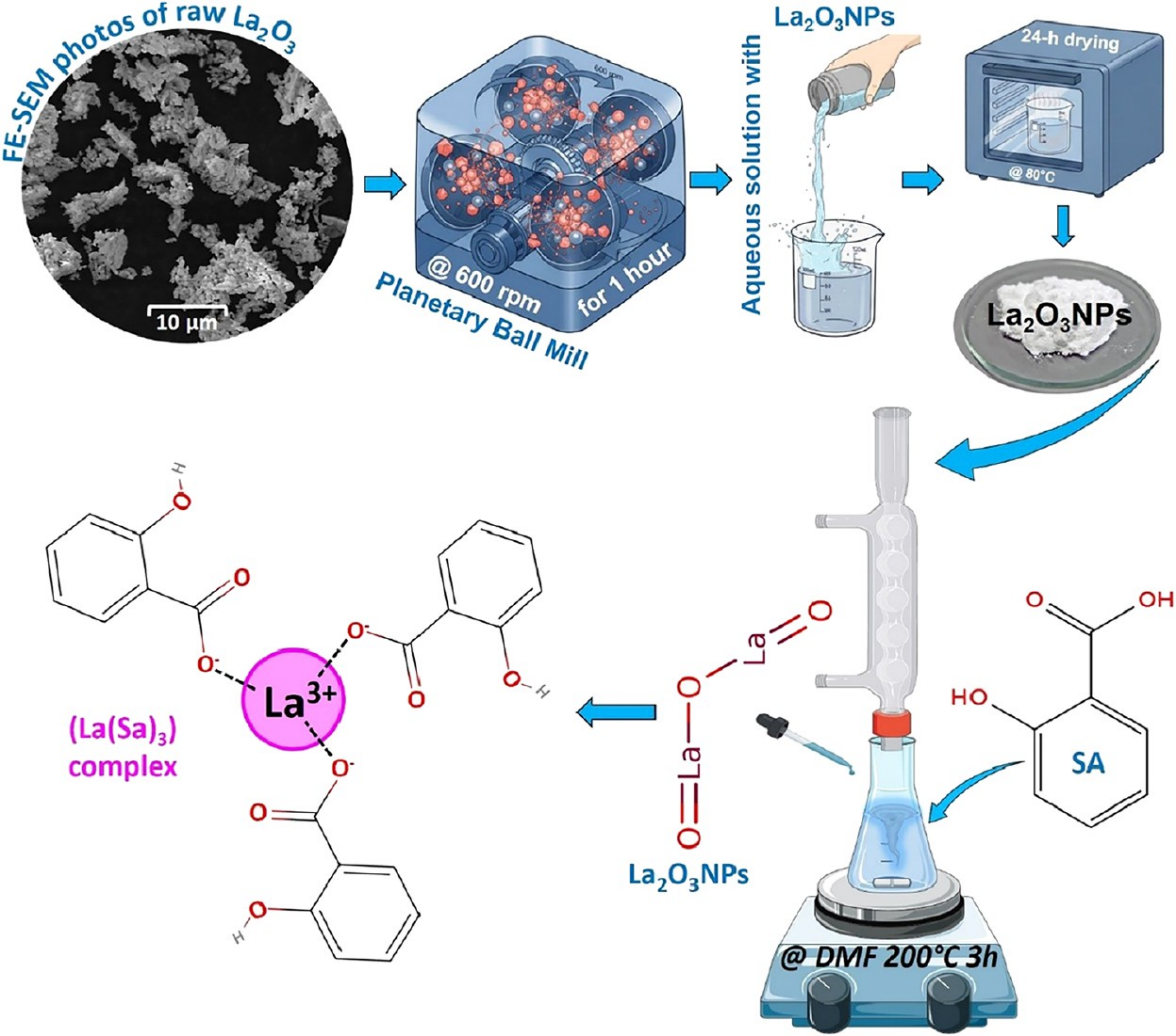
Schematic representations of the experimental steps involved in the synthesis of the La­(Sa)_3_ complex.

### 2.3 Characterizations of La_2_O_3_NPs and (La­(Sa)_3_) Complex

#### 2.3.1. Nuclear Magnetic Resonance Spectroscopy (NMR)

The newly obtained La­(Sa)_3_ complexes and La_2_O_3_NPs were structurally characterized using ^1^H NMR, ^13^C NMR, nuclear magnetic resonance spectroscopy (JEOL JNM-ECZ400S/L1, (400 MHz)). Measurements were performed by dissolving the sample in CDCl_3_, and chemical shifts (δ, ppm) were referenced to tetramethylsilane (TMS). Proton signals in ^1^H NMR spectra were interpreted on the basis of their multiplicity, chemical shift, and integration values. allowing differentiation between aromatic and aliphatic protons. In the ^13^C NMR analyses, carbon resonances were obtained in broadband decoupled mode. The positions and intensities of the signals in both spectra were used to elucidate the coordination environment and structural changes in the La_2_O_3_ NPs and La­(Sa)_3_ complexes. The temperature was controlled to within ± 0.1 K during the analyses.^31^ All spectral data were processed using MestReNova Lite software (2025, Mestrelab Research S.L.U.) and validated through comparison with literature reports.

#### 2.3.2 Fourier Transform Infrared (FTIR) Spectroscopy

For FTIR analysis, the La_2_O_3_ NPs and La­(Sa)_3_ complexes were dried at 25 °C prior to acquisition of their ATR-FTIR spectra. Spectral data were obtained using a Bruker α II FTIR spectrophotometer (Germany), with 128 scans collected across the 400–4000 cm^–1^ wavenumber range.^32^ The spectra of both materials were evaluated comparatively and the differences in their chemical structures and bonding properties were determined.

#### 2.3.3 TEM/FE-SEM Studies

The size and morphology of the synthesized nanoparticles were examined using a field-emission scanning electron microscope and scanning transmission electron microscope (FE-SEM/STEM; Tescan Mira3 XMU, Brno, Czechia). A small amount of each powder sample was placed onto an aluminum sample holder and subsequently coated with gold using a Q150T S Plus turbomolecular-pumped sputter coater (Sussex, U.K.). The morphology of the La_2_O_3_ NPs and La­(Sa)_3_ complexes was then analyzed under FE-SEM at an accelerating voltage of 10 kV, with a beam intensity of 10, under high-vacuum conditions (<9 × 10^–3^ Pa) in high-resolution mode. For TEM sample preparation,^33^ the powder samples were first dispersed in ethanol (CH_3_CH_2_OH) and ultrasonicated for 30 min to achieve homogeneous dispersion of the nanocrystalline material. A drop of the resulting suspension was then carefully pipetted onto TEM copper grid meshes. After drying under ambient conditions for 24 h, the deposited particles on the grids were directly examined using a field-emission electron gun (high-brightness Schottky emitter) equipped with a S-TEM detector. High-resolution micrographs were acquired in TEM bright-field mode, with environmental vibrations (<5 μm/s below 30 Hz) minimized and the working distance between the fixed sample and the objective lens maintained below 8 mm.

### 2.4 Molecular Docking Studies

Molecular docking studies were conducted to elucidate the interactions between the synthesized lanthanum salicylate compounds and the target microbial protein at the molecular level. Docking calculations were carried out using the *MzDOCK* software.^34,35^ The target protein structure was obtained from the Protein Data Bank (PDB) database with the code PDB ID: 3REM Figure 2.^36^ The protein structure was prepared prior to docking; H_2_O molecules, ions, and cocrystallized ligands present in the crystal structure were removed. Missing hydrogen atoms were added, appropriate partial charges were assigned to all atoms, and the structure underwent energy minimization. Three-dimensional geometries of the La­(Sa)_3_ complexes used as ligands were generated, and their geometries were optimized using molecular mechanics methods. The optimized ligand structures were then converted into a format suitable for docking.

**Figure 2:**
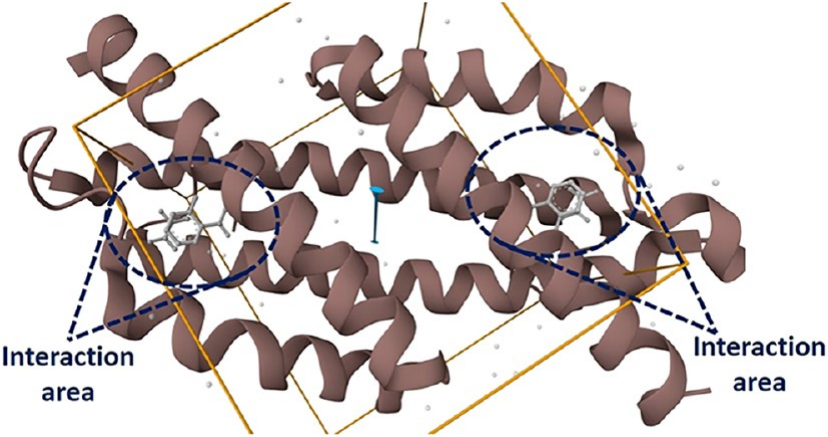
PDB ID: Representation of target sites in the 3REM molecule.

Docking simulations were performed within a grid box configured to fully encompass the active site of the target protein. The grid center coordinates were defined based on the binding pocket occupied by the cocrystallized ligand. Multiple conformations were generated for each ligand, and docking scores were calculated for each pose. The poses exhibiting the lowest binding energies were considered the most stable binding modes and were tabulated for comparison with the reference ligand Sa (salicylic acid).

To validate the docking methodology, a redocking procedure was performed, and the RMSD (Root Mean Square Deviation) values were calculated by comparing the predicted binding pose of the reference ligand with its crystallographic position. The resulting RMSD values were below 2.0 Å, confirming the reliability of the applied docking protocol.

### 2.5 *In Vitro* Antibacterial Studies

The *in vitro* antimicrobial activities of the synthesized La­(Sa)_3_ complexes and La_2_O_3_NPs were evaluated using the disk diffusion method.^37^ The reference microbial strains *Staphylococcus aureus* (Gram-positive, ATCC 29213), *Enterococcus faecalis*, (Gram-positive, ATCC 29212), *Enterococcus faecium* (Gram-positive, ATCC 1299), *Escherichia coli*, (Gram-negative, ATCC 25922), *Pseudomonas aeruginosa* (Gram-negative, ATCC 27853), *Klebsiella pneumonia* (Gram-negative, ATCC 700603) were obtained from the American Type Culture Collection (ATCC). The bacterial strains were activated by incubation on Mueller–Hinton Agar at 37 °C for 18–24 h. Microbial suspensions were prepared from fresh cultures in 0.85% NaCl solution and adjusted to the 0.5 McFarland standard. These standardized suspensions were uniformly spread onto the agar surfaces. Sterile 5 mm disks were impregnated with La_2_O_3_NPs and La­(Sa)_3_ complexes, and subsequently placed on the inoculated plates. Salicylic acid (salicylate) and broad-spectrum antimicrobial agents were used as positive controls, while disks containing only the solvent served as negative controls. In the antibacterial tests, standard antibiotic discs containing Vancomycin were used for *E. faecium* (VSEfm); Amoxicillin for *E. coli*, *S. aureus*, and *K. pneumoniae*; Amikacin for *P. aeruginosa*; and Linezolid for *E. faecalis*, respectively. The prepared plates were incubated at 37 °C for 24 h, after which the diameters of the inhibition zones were measured in millimeters. The results were expressed as the mean ± standard deviation of triplicate experiments (*n* = 3). The antimicrobial activity data were interpreted according to the criteria established by the European Committee on Antimicrobial Susceptibility Testing (EUCAST).

### 2.6 The Minimum Bactericidal Concentration (MBC) Assay

The minimum bactericidal concentration (MBC) of the tested compounds was determined using the broth microdilution method followed by subculturing on agar plates, in accordance with the Clinical and Laboratory Standards Institute (CLSI) guidelines.^38,39^ Briefly, bacterial strains were cultured on Mueller–Hinton agar (MHA) plates and incubated at 37 °C for 18–24 h to obtain fresh colonies. A bacterial suspension was prepared in sterile saline and adjusted to a turbidity equivalent to 0.5 McFarland standard (approximately 1 × 10^8^ CFU/mL). The suspension was further diluted in Mueller–Hinton broth (MHB) to achieve a final inoculum concentration of approximately 1 × 10^6^ CFU/mL. Serial 2-fold dilutions of the test compounds were prepared in MHB in sterile 96-well microtiter plates. Each well was inoculated with an equal volume of the standardized bacterial suspension. The plates were incubated at 37 °C for 24 h under aerobic conditions. After incubation, wells showing no visible bacterial growth were identified. To determine the MBC values, 1 μL aliquots from wells without visible turbidity were aseptically subcultured onto MHA plates and incubated at 37 °C for an additional 24 h. The lowest concentration of the compound that resulted in no colony growth on agar plates was recorded as the MBC, corresponding to *a* ≥ 99.9% reduction in the initial bacterial inoculum.^40^ A standard antibiotic was used as a (+) positive control, while broth containing the solvent without the test compound served as a (−) negative control. All experiments were performed in triplicate, and the results were expressed as mean values.

## 3 Results and Discussion

### 3.1 Powder Morphology and Microstructural Analyses

Examination of the FE-SEM photos presented in Figure 3 reveals that the La_2_O_3_NPs exhibit a heterogeneous morphology, consisting of a mixture of spherical/nonspherical and irregular particle forms. However, no well-defined or dominant particle shape can be clearly distinguished. Low-magnification images indicate that the particles exhibit a size distribution predominantly within the submicron range. In the high-magnification images, individual particles are observed to have sizes of approximately 100–300 nm, confirming that the milling process effectively reduced the La_2_O_3_ powders to the nanoscale. Concurrently, a pronounced tendency toward particle agglomeration is evident on the particle surfaces. Such behavior is characteristic of nano sized materials with high surface energy. The observed morphology appears to consist of numerous small crystallites, suggesting a polycrystalline structure. The agglomeration of the powder particles can be attributed to their high surface energy and the van der Waals attractive forces generated during the synthesis process.

**Figure 3:**
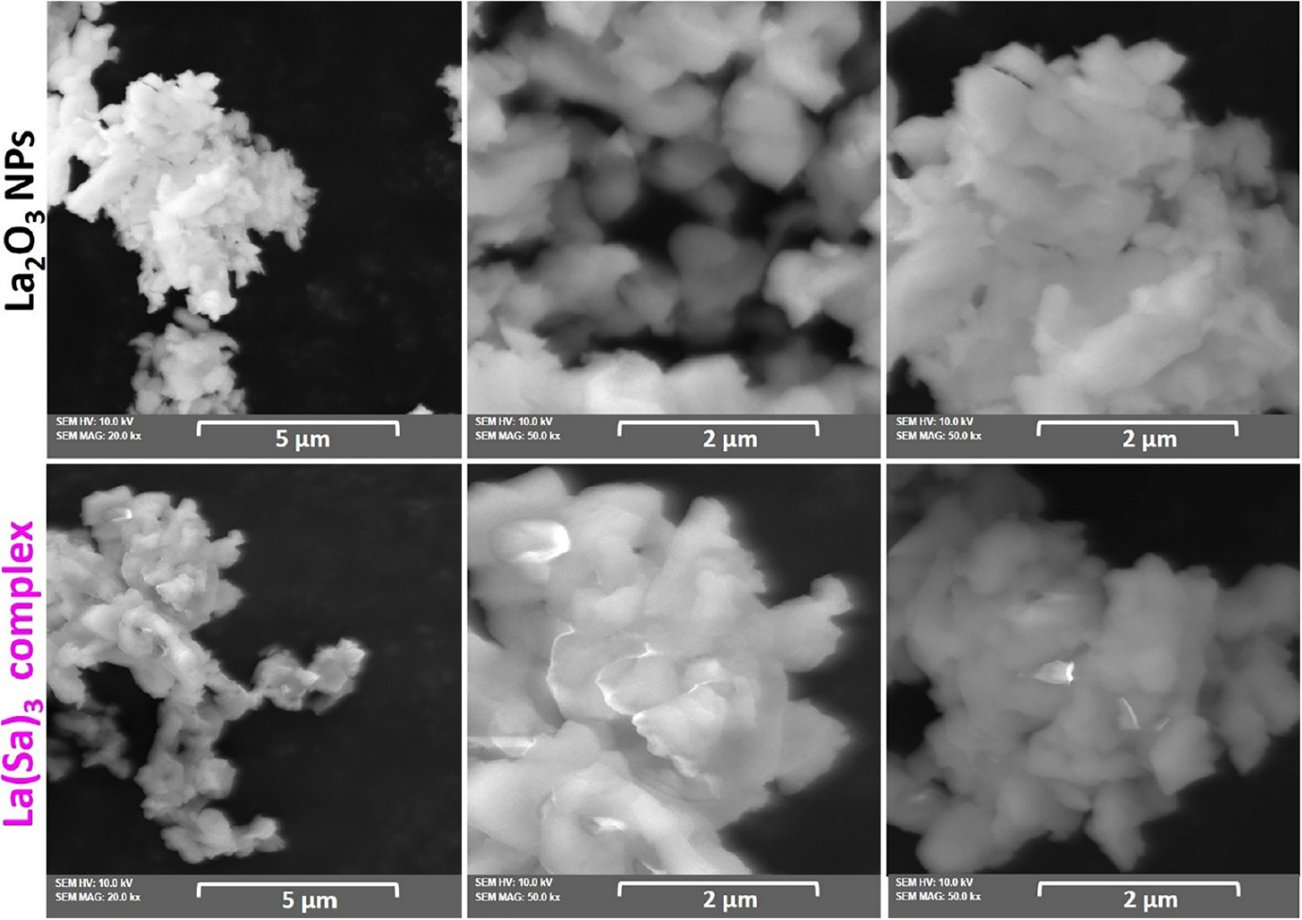
FE-SEM photographs of La_2_O_3_NPs and La­(Sa)_3_ complex.

As observed in the micrographs of the La­(Sa)_3_ complex particles also exhibit an agglomerated morphology; however, these agglomerates are comparatively more open and sponge-like than the dense clusters observed in the La_2_O_3_NPs samples. This difference can be attributed to the influence of salicylic acid ligand coordination on the particle growth mechanism. High-magnification micrographs further reveal that the La­(Sa)_3_ complex particles possess more sharply defined layered boundaries and display a rougher surface topography than La_2_O_3_NPs. Such morphological features are characteristic of coordination polymer structures formed through interactions between the salicylic acid ligand and the metal ions and may influence the morphology of the resulting oxide following thermal treatment.^41,42^ While the FE-SEM photos of both samples exhibit closely aligned morphological profiles, the discrete variations in their surface topographies allow for further comment on the distinct physical properties characterizing each sample.

Figure 4a displays S-TEM images of the La_2_O_3_NPs and La­(Sa)_3_ complex synthesized in this experimental study, allowing a more precise determination of the morphologies of both powders. S-TEM micrographs clearly reveal the morphological differences between the La­(Sa)_3_ complex and the La_2_O_3_NPs. Following high-energy wet milling, the La_2_O_3_NPs generally exhibit spherical or hemispherical morphologies, with an average particle size of approximately 53.13 ± 16.51 nm (Figure 4b). In contrast, a significant morphological transformation was observed in the La­(Sa)_3_ complex obtained via solid–liquid phase reaction in *N,N*-dimethylformamide under reflux conditions.

**Figure 4:**
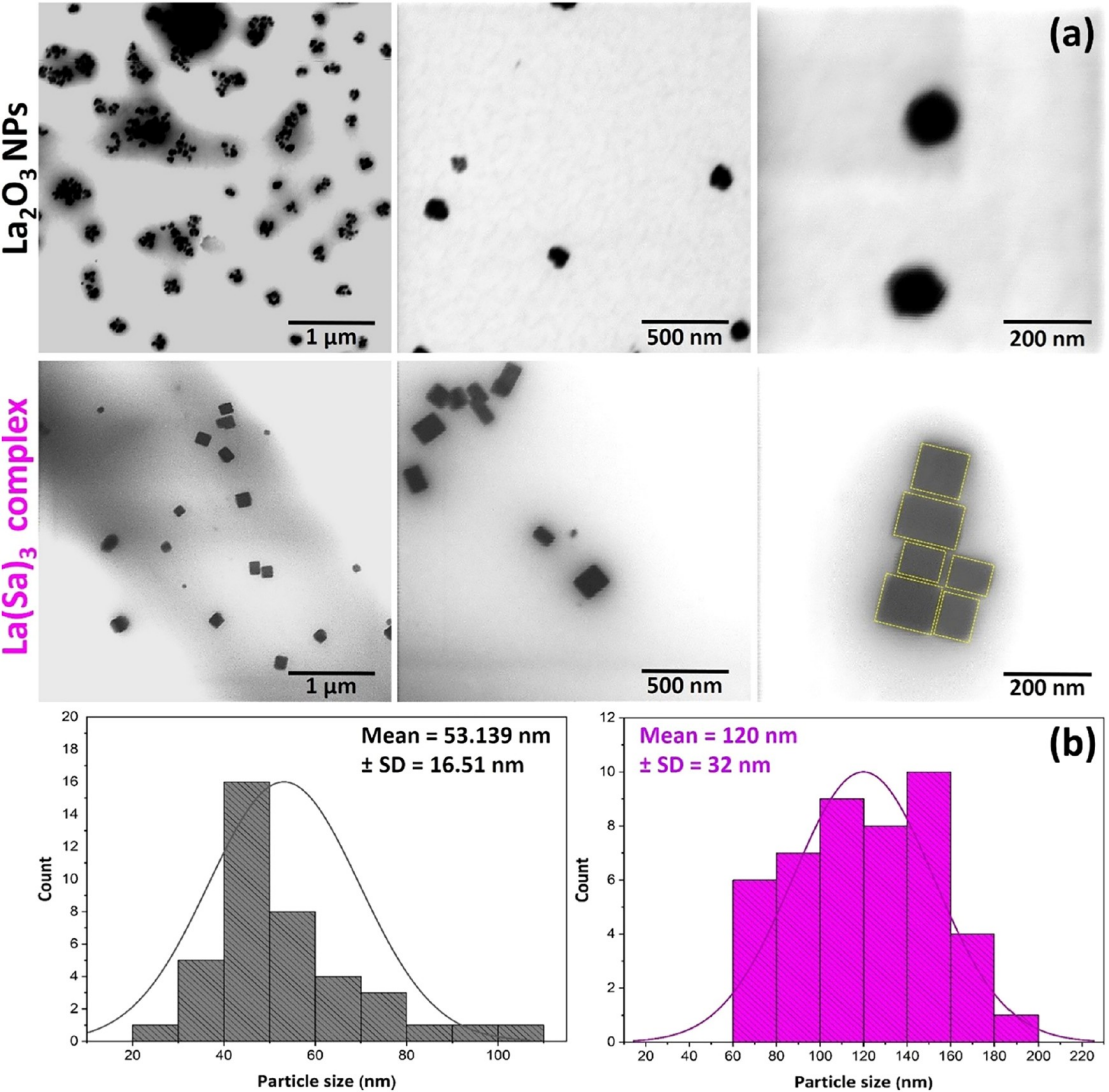
(a) High magnification (a) S-TEM photographs and (b) particle size distribution histogram of La_2_O_3_NPs (black) and La­(Sa)_3_ complex (pink).

The morphology of the complex evolved into a cubic geometry, distinctly different from the spherical morphology of the La_2_O_3_NPs. The average size of these cubic particles was determined to be approximately 120 ± 32 nm (Figure 4b), indicating a moderate increase in particle size during the reaction process. The transformation from spherical La_2_O_3_NPs to cubic La­(Sa)_3_ complex particles can be primarily attributed to coordination chemistry and thermodynamically controlled crystal growth. During synthesis, the La_2_O_3_NPs undergo a dissolution–reprecipitation process in the presence of salicylic acid. The La^3+^ ions released from La_2_O_3_ subsequently coordinate with the salicylic acid ligands, leading to the formation of the La­(Sa)_3_ complex. The intrinsic crystallographic structure of the La­(Sa)_3_ complex—defined by the stable coordination arrangement of ligands around the La^3+^ center—thermodynamically favors the development of a cubic or pseudocubic lattice.^43^ The elevated reaction temperature (200 °C) and controlled reflux conditions drive the system toward thermodynamic control rather than kinetic control. This shift promotes the formation of a stable and uniform crystal morphology (cubic structure) corresponding to the lowest free energy state of the complex.^44–46^ Consequently, the observed morphological change arises directly from ligand coordination, which induces the formation of a new crystalline phase La­(Sa)_3_, rather than from the initial La_2_O_3_NPs acting as a structural template.

### 3.2 Fourier-Transform Infrared Spectroscopy Analyses (FTIR)

The Fourier transform infrared (FTIR) spectrum was recorded for the as-prepared samples. The FTIR spectra of the La_2_O_3_NPs and the La­(Sa)_3_ complex are presented in Figure 5. In the FTIR spectrum of the La_2_O_3_NPs, the broad bands observed at 3607 and 3449 cm^–1^ confirm the presence of O–H stretching vibrations, which are attributed to adsorbed moisture on the surface of the samples.^47^ The peaks at 1477 cm^–1^ and the distinct band at 1386 cm^–1^ originate from the asymmetric and symmetric stretching vibrations of COO^–^ functional groups, respectively.^48^ Another band observed at 856 cm^–1^ is associated with C–O bending vibrations. The broad band at 777 cm^–1^ and the sharp peak at 631 cm^–1^ are assigned to La–O stretching vibrations, whereas the weak band at 463 cm^–1^ corresponds to La–O bending vibrations. The presence of these characteristic La–O stretching and bending vibration bands therefore confirms the existence of the Lanthanum oxide phase in the nano powder samples.^49^

**Figure 5:**
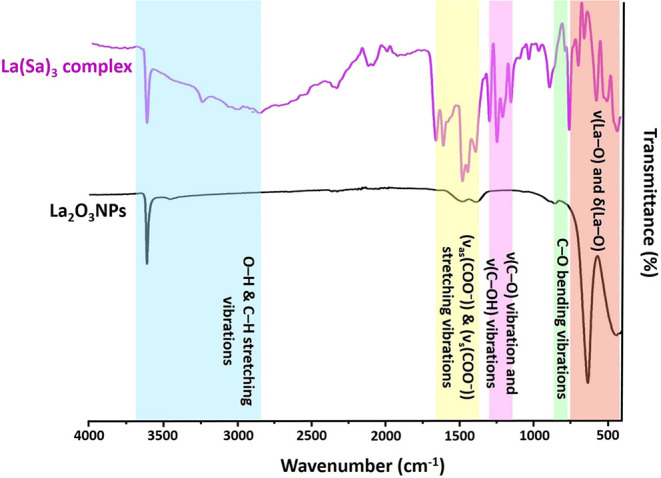
FTIR spectra of La_2_O_3_NPs and the La­(Sa)_3_ complex.

The formation of the La­(Sa)_3_ complex was further confirmed by FTIR spectral analysis in comparison with La_2_O_3_NPs. The FTIR spectrum of the La­(Sa)_3_ sample exhibited characteristic vibrational bands at 3607 cm^–1^, 3235 cm^–1^, and in the range of 3000–2849 cm^–1^, which were assigned to O–H and C–H stretching vibrations, respectively. The C=O (COO^–^) asymmetric and symmetric stretching were assigned to IR peaks observed at 1656 cm^–1^ and 1389 cm^–1^, respectively.^50^ Furthermore, the IR spectrum of the La­(Sa)_3_ complex exhibits multiple peaks in the range of 1578–1607 cm^–1^, which are attributed to C=C stretching vibrations of the phenolic moieties, while the distinct bands observed at 1578 and 1389 cm^–1^ correspond to the asymmetric (ν_as_(COO^–^)) and symmetric (ν_s_(COO^–^)) stretching vibrations of the carboxylate groups, respectively.^51^ The C–C stretching vibrations were observed in the range of 1442–1511 cm^–1^. Additionally, the ν­(C=N) stretching vibration detected at 1442 cm^–1^ in the La­(III) complex suggests coordination between the nitrogen atoms of the phen ligand and the La­(III) center. The O–H (phenolic) bending vibration was assigned to the IR band observed at 1330 cm^–1^. The COO^–^ (C–O) stretching vibration and the C–OH (phenolic) stretching vibrations were attributed to the IR bands at 1294 cm^–1^ and in the range of 1150–1244 cm^–1^, respectively. The vibrational bands observed in the range of 755–654 cm^–1^ were assigned to =C–H bending vibrations.^50,52^

The shoulder bands observed at 431 and 501 cm^–1^, which are attributed to La–O asymmetric stretching vibrations and chelate ring bending vibrations, respectively, further indicate that all C=O groups participate in coordination with the rare-earth La^3+^ ion.^51^

### 3.3 Nuclear Magnetic Resonance Analysis (^13^C NMR - ^1^H NMR)

#### 3.3.1 La­(Sa)_3_ Complex Analyses

The chemical synthesis of La­(Sa)_3_ complex yield was 78%. ^1^H NMR and ^13^C NMR chromatogram of the La­(Sa)_3_ complex is presented in Figures 6 and 7 respectively. ^13^C NMR (150 MHz, DMSO): δ 39.81 (C-7), δ 39.36 (C-6), δ 39.08 (C-5), δ 39.02 (C-4), δ 38.97 (C-3), δ 38.79 (C-2). ^1^H NMR (400 MHz, DMSO): δ 6.03–5.42 (m, H-3, H-4, olefinic CH), δ 2.47 (s, H-7, CH_2_–X), δ 1.02 (t/s, H-9, CH_3_). In the ^1^H NMR spectrum, olefinic protons appeared as multiples in the range of δ 5.42–6.03 ppm, while aliphatic and methyl protons were observed at δ 2.47 and 1.02 ppm, respectively. Compared to the free ligand, all proton signals exhibited slight upfield shifts, indicating a shielding effect induced by lanthanum coordination. The ^13^C NMR spectrum showed carbon resonances clustered between δ 38.79 and 39.81 ppm, consistent with aliphatic and functional carbons influenced by metal coordination. The observed upfield shifts and signal broadening confirm successful metal–ligand interaction and formation of the La­(Sa)_3_ complex.

**Figure 6:**
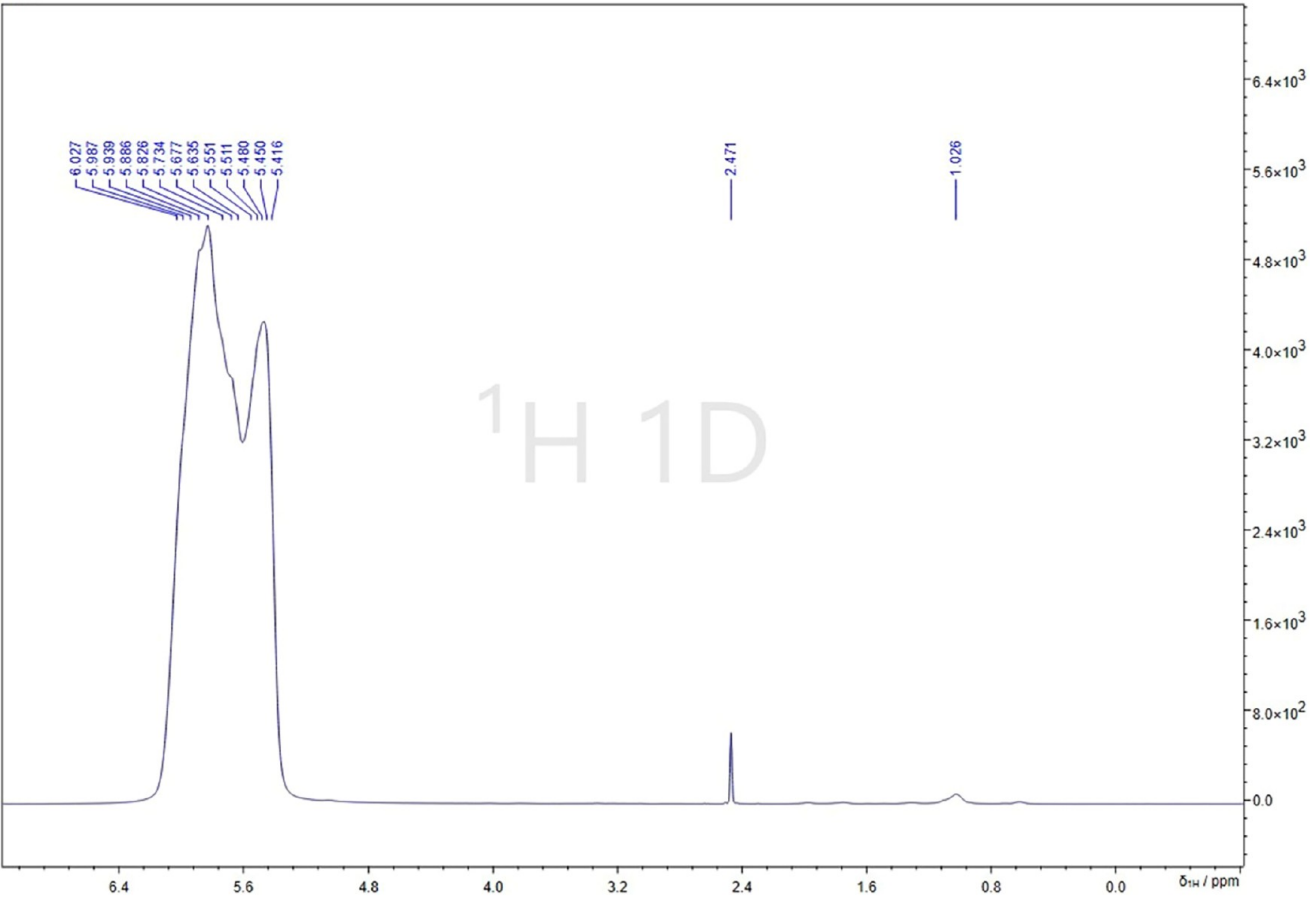
^1^H NMR chromatogram of the La­(Sa)_3_ complex.

**Figure 7:**
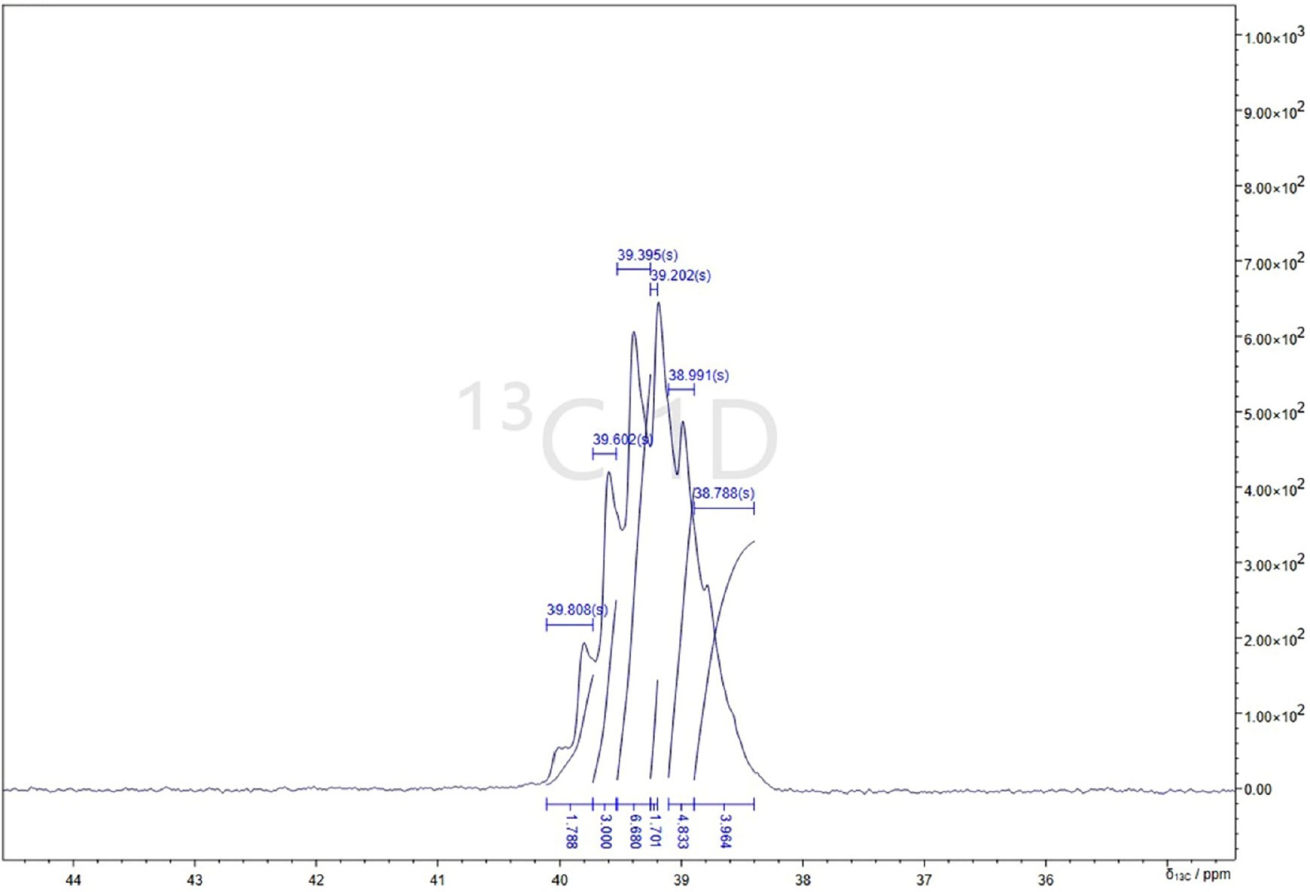
^13^C NMR chromatogram of the La­(Sa)_3_ complex.

The concentration of signals belonging to olefinic protons in the 5.4–6.0 ppm range confirms the presence of a conjugated double bond system in the compound. Due to the coordinative effect of the La^3+^ ion, these protons were observed to shift slightly toward higher fields compared to their free ligand counterparts reported in the literature. This can be explained by lanthanum redistributing the electron density, thereby creating a shielding effect on the protons.

In the ^1^H NMR spectrum, signals belonging to aromatic protons were found to cluster in the 5.416–6.027 ppm range. These protons, typically observed around 7.8–8.2 ppm in free salicylic acid, were found to shift to higher fields following lanthanum coordination. Furthermore, the carboxylic acid proton was not observed in the spectrum; this strongly supports the coordination of the carboxylate group (−COO) with the metal ion (Figure 6).

The clustering of carbon signals within a narrow range (δ 38.8–39.8 ppm) in the ^13^C NMR spectrum indicates that the lanthanum center significantly affects the magnetic environment around the carbon. Due to lanthanum’s high atomic number and paramagnetic contribution potential, a shift toward higher fields and signal broadening were observed in the carbon signals. This feature supports the successful metal–ligand coordination and the presence of carbon atoms in similar electronic environments. This upfield shift effect is due to the La^3+^ ion increasing the electron density on the ligand because of its high electrostatic field, and the protons in the aromatic ring becoming magnetically less deshielded. Up field shifts in both ^13^C NMR and ^1^H NMR signals following metal–oxygen coordination have also been reported in the literature for similar La­(Sa)_3_ complexes. This confirms that the synthesized structure is a salicylate complex coordinated to the lanthanum center via the carboxylate oxygen atoms (Figure 7).^53^

When ^1^H NMR and ^13^C NMR spectra are evaluated together, it is observed that lanthanum coordination creates a significant magnetic shielding effect on the ligand skeleton. The high field shifts observed particularly in olefinic protons and aliphatic carbons confirm that the metal–ligand interaction has been successfully achieved and that the synthesized complex is consistent with the targeted structure. The differences between the ^1^H NMR and ^13^C NMR spectra of SA and the La­(Sa)_3_ complex are presented in Table 1.

**Table 1:** Data on the ^13^C-NMR and ^1^H-NMR Differences between SA and La­(Sa)_3_ Complex

functional groups	free ligand δ (ppm)^a^	La(Sa)_3_ δ (ppm)	direction of slip	description
olefinic H (H-olefinic)	5.6–6.2	5.42–6.03	↑ area	La^3+^ coordination → shielding
aliphatic H (H-α)	2.6	2.47	↑ area	metal–ligand interaction
methyl H (CH_3_)	1.15	1.02	↑ area	increase in electron density
aliphatic C (C-α)	40–42	38.79–39.81	↑ area	paramagnetic effect
functional C	wide distribution	narrow gap		similar electronic environment

a The chromatogram of SA was obtained using the Spectra Base Database.^54^

### 3.4 Antibacterial Studies

The antibacterial activities of La_2_O_3_NPs, SA, and La­(Sa)_3_ complexes were evaluated against selected Gram-positive and Gram-negative bacterial strains using the Kirby–Bauer disk diffusion method. The schematic illustration of the experimental steps associated with the Kirby–Bauer disk diffusion method was presented in Figure 8a, while the inhibition zone diameters (mm) obtained using ATCC reference strains were summarized in Figures 8b and 9. La­(Sa)_3_ complex exhibited notable antibacterial activity against most tested microorganisms. The highest inhibition zones were observed against *E. faecium* (VSEfm) (30 mm), *E. coli* (24 mm), and *S. aureus* (22 mm). Moderate activity was detected against *P. aeruginosa* (20 mm) and *E. faecalis* (EF) (11 mm), while weak inhibition was observed against *K. pneumoniae* (8 mm). SA showed comparatively lower antibacterial activity than La­(Sa)_3_ complex across all tested strains. Inhibition zones ranged from 10–18 mm for Gram-positive bacteria and 0–12 mm for Gram-negative bacteria. Notably, SA exhibited no detectable antibacterial activity against *K. pneumoniae*. In contrast, La_2_O_3_NPs did not exhibit any antibacterial activity against any of the tested microorganisms, as no inhibition zones were observed. Positive controls demonstrated expected antibacterial activity, while negative controls showed no inhibition, confirming the validity of the experimental conditions.

**Figure 8:**
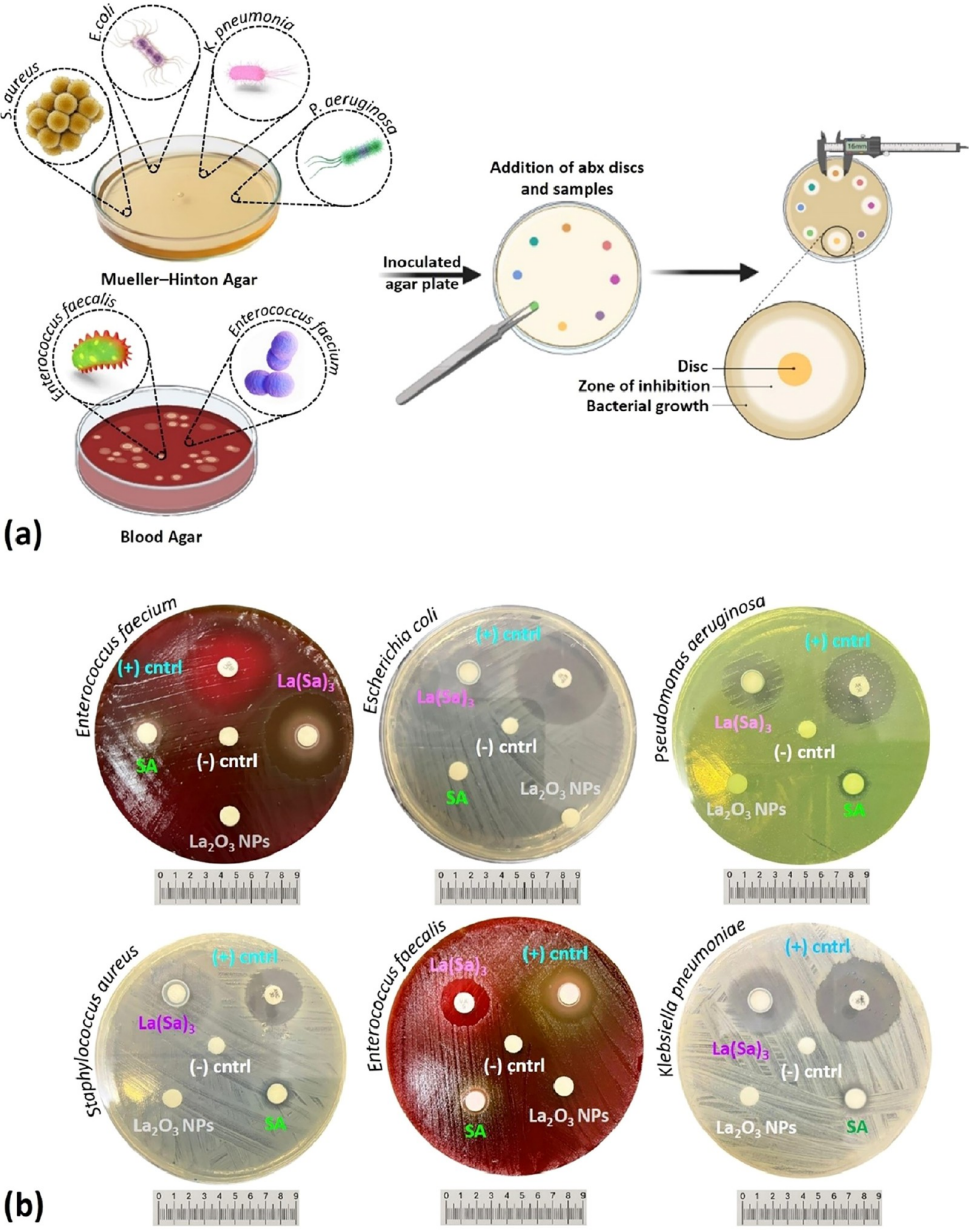
(a) Schematic illustration of the experimental steps involved in the Kirby–Bauer disk diffusion method, (b) representative photographs of inhibition zone diameters on agar plates obtained using ATCC reference strains.

**Figure 9:**
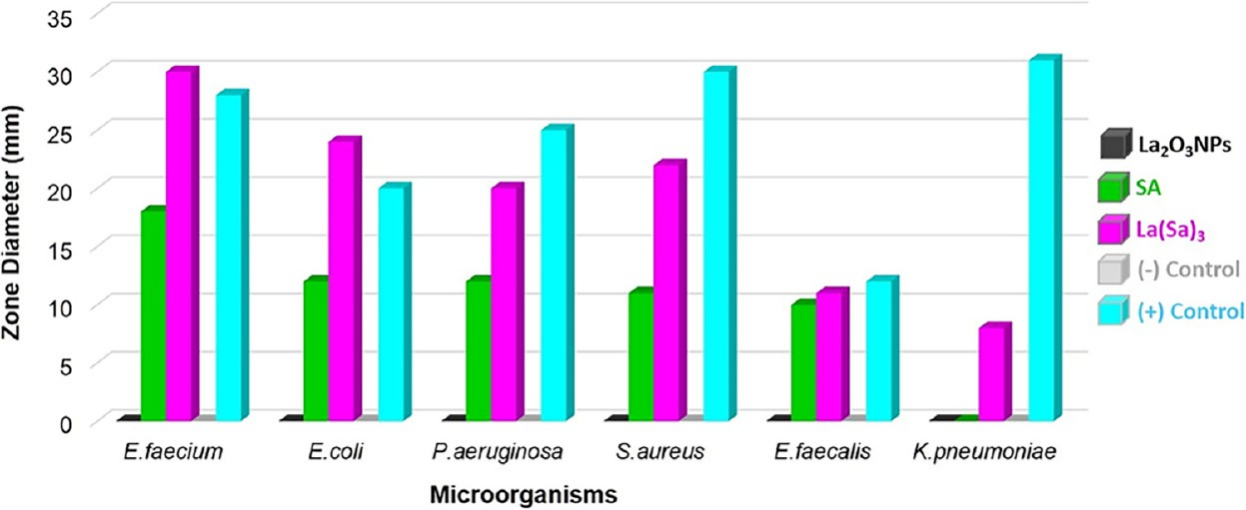
Antibacterial activity of La_2_O_3_NPs, SA, and La­(Sa)_3_ complex samples.

The enhanced antibacterial activity observed for the La­(Sa)_3_ complex compared to SA alone can be attributed to multiple synergistic mechanisms arising from lanthanum coordination. Complexation with La^3+^ ions is known to alter the physicochemical properties of organic ligands, including increased lipophilicity, improved membrane permeability, and enhanced stability under physiological conditions. These changes facilitate more efficient interaction of the La­(Sa)_3_ complex with bacterial cell membranes, promoting membrane destabilization and increased intracellular penetration. Furthermore, the presence of the lanthanum center may enable electrostatic interactions with negatively charged components of the bacterial cell envelope, such as phospholipids and teichoic acids in Gram-positive bacteria, as well as lipopolysaccharides in Gram-negative bacteria. This interaction can disrupt membrane integrity, leading to leakage of intracellular contents and eventual cell death. In addition, metal ligand complexes have been reported to interfere with essential bacterial enzymes by binding to active-site residues or metal-dependent catalytic motifs, thereby inhibiting critical metabolic pathways. The lack of antibacterial activity observed for La_2_O_3_NPs alone suggests that the biological effect is not solely driven by the inorganic component, but rather by the coordinated structure of the La­(Sa)_3_ complex. In this context, SA acts not only as an antibacterial pharmacophore but also as a carrier ligand that facilitates the biological availability of the lanthanum ion. The significantly larger inhibition zones obtained for La­(Sa)_3_ therefore indicate a synergistic effect between the lanthanum center and the salicylate ligand, resulting in a more potent and broad-spectrum antibacterial response than that of the free ligand.

### 3.5 The Minimum Bactericidal Concentration (MBC) Assay

The bactericidal activity of La_2_O_3_NPs, SA, and La­(Sa)_3_ complexes was evaluated based on MBC determinations following MIC assays. MBC values were interpreted in relation to MIC results to distinguish between bactericidal and bacteriostatic effects. For La_2_O_3_NPs, no bactericidal effect was observed against the tested bacterial strains within the studied concentration range. Although partial growth inhibition was detected at higher concentrations in some strains, viable colonies were recovered upon subculturing from MIC wells, indicating that La_2_O_3_NPs exhibited no measurable MBC under the tested conditions. This suggests a predominantly inactive or weak antibacterial profile for La_2_O_3_NPs when applied alone. In contrast, SA demonstrated clear bactericidal activity against both Gram-positive and Gram-negative bacteria. The MBC values were generally close to the corresponding MIC values, particularly for *S. aureus*, *E. faecium* (VRE and VSE), and *E. coli* strains, indicating a bactericidal mode of action. For most strains, the MBC/MIC ratio was ≤ 2, supporting the conclusion that SA effectively reduced bacterial viability rather than merely inhibiting growth. Notably, the La­(Sa)_3_ complex exhibited enhanced bactericidal efficacy compared to SA alone. For several tested strains, including *S. aureus*, *E. faecium* (VRE), and *E. coli* (ATCC), MBC values were either identical to or only one dilution higher than the MIC values. This narrow MBC/MIC ratio strongly indicates a potent bactericidal effect. Moreover, La­(Sa)_3_ maintained bactericidal activity against problematic Gram-negative pathogens such as *P. aeruginosa* and *K. pneumoniae*, where SA alone required higher concentrations to achieve complete bacterial killing. Overall, the MBC findings demonstrate that while La_2_O_3_NPs alone lacks bactericidal activity, complexation with SA significantly enhances antibacterial performance. The improved bactericidal effect of La­(Sa)_3_ may be attributed to synergistic interactions between the lanthanum ion and the salicylate ligand, potentially facilitating increased membrane permeability or intracellular targeting. These results highlight La­(Sa)_3_ as a promising bactericidal agent with broad-spectrum activity. The MBC results for La_2_O_3_ NPs, SA, and the La­(Sa)_3_ complex are shown in Figure 10. The pronounced bactericidal activity of the La­(Sa)_3_ complex observed in the MBC assays can be attributed to the synergistic effect between the lanthanum ion and the salicylate ligand. The close MBC/MIC ratios obtained for several strains indicate that La­(Sa)_3_ does not merely inhibit bacterial growth but induces irreversible cellular damage. This effect is likely associated with enhanced membrane permeability and intracellular accumulation facilitated by metal–ligand coordination. Once internalized, the La^3+^ center may interfere with essential bacterial enzymes or metal-dependent biochemical pathways, leading to loss of metabolic activity and cell death. In contrast, the absence of measurable MBC values for La_2_O_3_NPs alone suggests that effective bactericidal action requires the coordinated La­(Sa)_3_ structure rather than the inorganic component by itself.^55^

**Figure 10:**
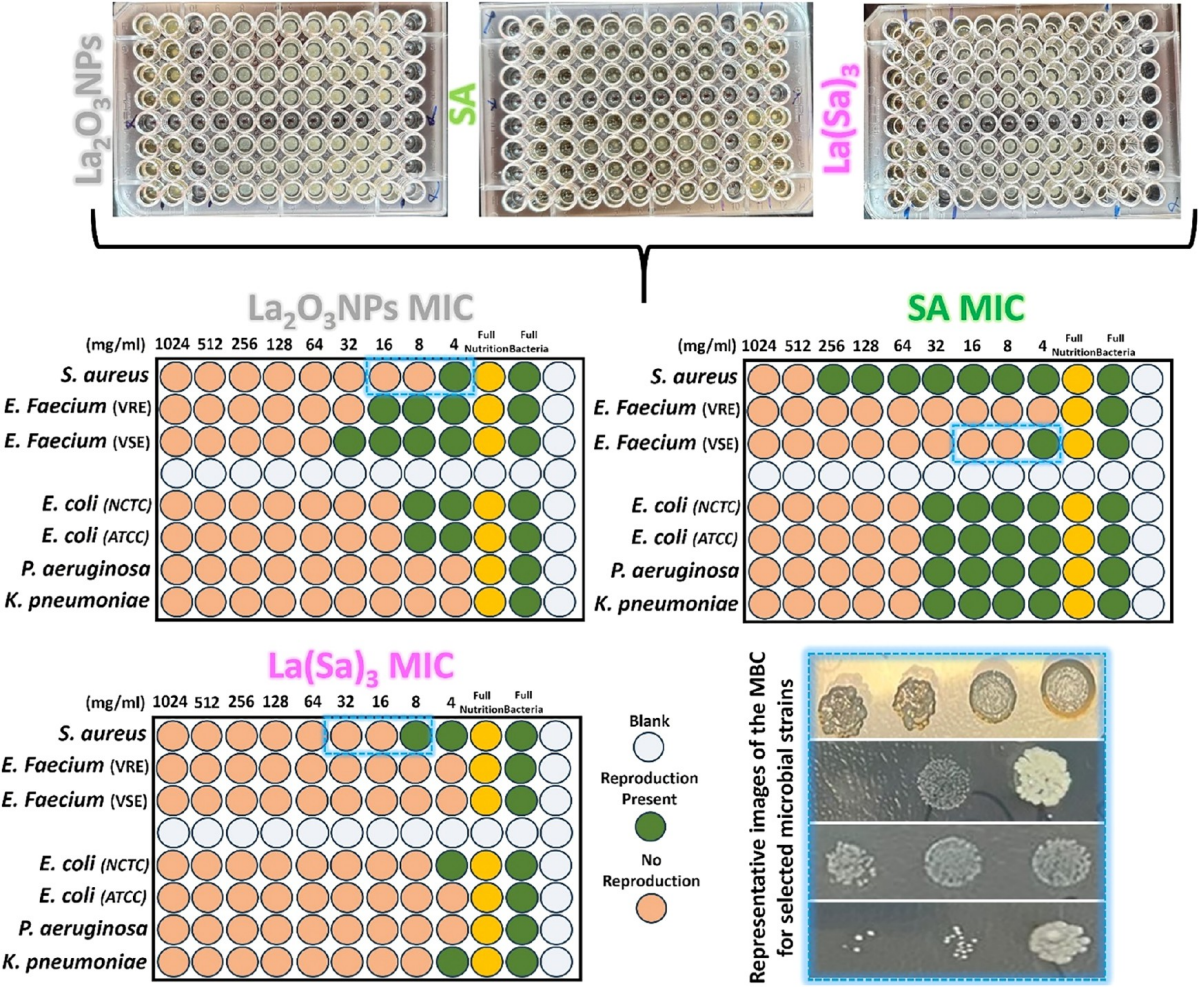
MBC results of La_2_O_3_NPs, SA, and La­(Sa)_3_ complex. Representative photographs of bacteria inoculated into microtiter plates at different dilutions after incubation for 24 ± 2h at 37 °C; Schematic images illustrating the growth behavior of materials including La_2_O_3_NPs, SA, and La­(Sa)_3_ complex in microtiter plates; and stereomicroscopic images of wells showing the absence of bacterial colony formation after the tapping procedure and subsequent incubation for 24 ± 2 h at 37 °C (regions indicated by blue dashed lines).

### 3.6 Molecular Docking Studies

As shown in Figure 11, in 3D interactions, the La­(Sa)_3_ molecule captures more interactions from the target area than the SA molecule alone. A comparison of the lowest-energy binding positions, considered the most stable attachment modes, with the reference ligand (SA) is presented in Table 2, while higher docking scores relative to SA are presented in Table 3. Table 3 clearly shows that the newly synthesized La­(Sa)_3_ complex has a higher (better) docking score compared to the SA compound used as a coligand. This result demonstrates that lanthanum coordination enhances the interaction with the target protein and significantly increases the binding affinity. Furthermore, the fact that the RMSD values obtained in all calculations are below 2 Å demonstrates the reliability of the molecular docking analyses performed and confirms that the biological activity results obtained *in vitro* are consistent with the *in silico* data. This consistency is considered an important indicator of success, demonstrating that the biological potential of the synthesized lanthanum La­(Sa)_3_ is supported by both experimental and computational methods.

**Figure 11:**
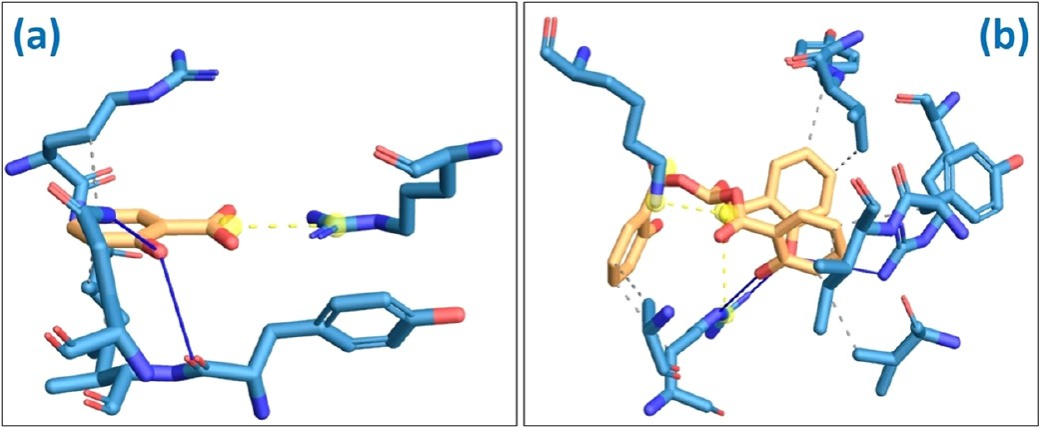
(a) 3D interaction between the SA molecule and PDB ID: 3REM, (b) 3D interaction between the La­(Sa)_3_ molecule and PDB ID: 3REM.

**Table 2:** Docking Datas of SA Molecule and La­(Sa)_3_ Molecule

**compounds**	**docking score**
SA (redocking refarance compound)	–5.8 kcal/mol
La(Sa)_3_	–7.7 kcal/mol
RMSD: 1.072 Å	

**Table 3:** Table of Similarities between La­(Sa)_3_ and *in Silico* Reference Molecule SA Interactions

**residue**	**SA interaction**	**La(Sa)**_**3**_ i**nteraction**	**SA distance**	**lig distance**	**distance diff**.
Arg31	salt-bridge	H-bond	3.45	3.6	0.15
Tyr86	H-bond	hydrophobic	3.57	3.64	0.07
Arg53	hydrophobic	hydrophobic	3.94	3.76	–0.18
Arg53	hydrophobic	H-bond	3.94	2.57	–1.37
Arg53	hydrophobic	H-bond	3.94	1.81	–2.13
Arg53	hydrophobic	salt-bridge	3.94	4.59	0.65
Ile87	hydrophobic	hydrophobic	3.39	3.87	0.48

When examining Table 4 and the data pertaining to Table 4, it is observed that the interactions established by SA and La­(Sa)_3_ complex with the target region exhibit significant differences in terms of both diversity and interaction quality. The La­(Sa)_3_ complex establishes a greater number and stronger interactions compared to the SA compound, indicating an increased ability to bind to the target region and providing an advantage in terms of structure–activity relationship. In particular, the positive reflection of these additional interactions of the complex on *in vitro* biological activity results demonstrates that the computational data obtained are consistent with experimental findings. These results support the notion that lanthanum coordination plays an important role in enhancing the biological activity of the compound by enriching its interaction profile. The molecular docking results provide quantitative evidence supporting the enhanced antibacterial activity of the La­(Sa)_3_ complex. Compared to SA, La­(Sa)_3_ exhibited a markedly higher docking score with a more negative binding energy, indicating a stronger and more stable interaction with the target protein. In addition, the La­(Sa)_3_ complex formed a greater number of interactions, including hydrogen bonds, hydrophobic contacts, and salt bridges, with shorter interaction distances. The increased number and diversity of these interactions contribute to improved binding affinity and stabilization of the ligand protein complex. The higher absolute docking score and enriched interaction profile of La­(Sa)_3_ are therefore consistent with its superior *in vitro* antibacterial performance, suggesting that stronger target engagement plays a key role in its enhanced biological activity.

**Table 4:** Properties of the Bonds between La­(Sa)_3_ and 3REM

**residues**	**interaction type**	**distance**
Tyr34	hydrophobic	3.55
Val35	hydrophobic	3.41
Ala50	hydrophobic	3.9
Arg53	hydrophobic	3.76
Ile83	hydrophobic	3.88
Tyr86	hydrophobic	3.64
Ile87	hydrophobic	3.87
Arg31	H-bond	3.6
Arg53	H-bond	2.57
Arg53	H-bond	1.81
Lys42	salt-bridge	4.05
Lys42	salt-bridge	3.33
Arg53	salt-bridge	4.59

## 4 Conclusion

In this experimental study, a novel La­(Sa)_3_, was successfully synthesized via a controlled coordination reaction between SA and La_2_O_3_NPs and comprehensively characterized using spectroscopic and microscopic techniques. Structural analyses confirmed the effective coordination of salicylate ligands to the La^3+^ center, accompanied by a pronounced transformation in particle morphology from spherical La_2_O_3_NPs to a well-defined cubic La­(Sa)_3_ structure. These findings demonstrate that ligand coordination plays a decisive role not only in chemical structure formation but also in governing the physicochemical properties of the resulting complex. The biological evaluations revealed that the La­(Sa)_3_ complex exhibits significantly enhanced antibacterial activity compared to both the free salicylic acid ligand and La_2_O_3_NPs alone. Disk diffusion and MBC assays demonstrated that La­(Sa)_3_ possesses broad-spectrum antibacterial and bactericidal activity against both Gram-positive and Gram-negative bacterial strains. The close MBC/MIC ratios observed for several clinically relevant pathogens indicate that the complex induces irreversible bacterial damage rather than merely exerting a bacteriostatic effect. The absence of comparable activity for La_2_O_3_NPs highlights that effective antibacterial performance arises from the coordinated metal ligand architecture rather than from the inorganic component alone. The enhanced antibacterial efficacy of La­(Sa)_3_ can be attributed to a synergistic mechanism involving increased membrane permeability, stronger electrostatic interactions with bacterial cell envelopes, and improved intracellular accessibility mediated by lanthanum coordination. This mechanistic interpretation is strongly supported by molecular docking studies, which demonstrated that La­(Sa)_3_ exhibits a substantially higher absolute docking score and a richer interaction profile with the target microbial protein compared to SA. The increased number of hydrogen bonds, hydrophobic interactions, and salt bridges, together with shorter interaction distances, indicate stronger and more stable target engagement, which correlates well with the experimentally observed antibacterial potency. Overall, the strong agreement between *in vitro* antibacterial assays and *in silico* docking analyses confirms that lanthanum coordination significantly enhances the biological performance of SA. The findings of this study highlight La­(Sa)_3_ as a promising metal based antibacterial candidate and underscore the potential of La­(Sa)_3_ complexes as a versatile platform for the rational design of new antimicrobial agents. Future studies focusing on cytotoxicity, pharmacokinetics, and *in vivo* efficacy will be essential to further advance this class of compounds toward biomedical applications.
